# Endothelial autophagy blockade fosters anti-cancer immunity

**DOI:** 10.1038/s44321-023-00005-x

**Published:** 2023-12-20

**Authors:** Giulia Villari, Guido Serini

**Affiliations:** 1 Department of Oncology, University of Torino School of Medicine, Candiolo, TO Italy; 2 Candiolo Cancer Institute - Fondazione del Piemonte per l’Oncologia (FPO) Istituto di Ricovero e Cura a Carattere Scientifico (IRCCS), Candiolo, TO Italy

**Keywords:** Autophagy & Cell Death, Cancer, Vascular Biology & Angiogenesis

## Abstract

G. Serini and G. Villari discuss the recent study by Verhoeven et al, in which autophagy is identified as a tumor vascular anti-inflammatory mechanism that impairs melanoma anti-tumor immunity.

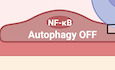

## Teaser

The discovery of immune checkpoints, consisting of transmembrane ligand-receptor protein pairs that negatively regulate the CD8^+^ T-cell-mediated immune response by antigen-presenting cells (APCs) and by cancer cells, has enabled a fundamental advancement of cancer therapies (Korman et al, 2022). Indeed, harnessing these homeostatic control mechanisms to their own advantage, tumor cells manage to defend themselves from the attack of the immune system, and immune checkpoint blockade (ICB) has proven to be an overwhelmingly successful antitumor therapeutic approach (Korman et al, 2022).

Presumably due to its high burden of mutations and neoantigens, cutaneous melanoma, derived from the neoplastic transformation of skin melanocytes, is one of the cancer histotypes that has benefited most from ICB-based treatments (Kalaora et al, 2022). At least in part in response to interferon γ (IFN γ) released by CD8^+^ T cells (Taube et al, 2012), melanoma cells overexpress on their surface programmed death ligand 1 (PD-L1) and PD-L2, two prototypical immune checkpoint transmembrane ligands that interact with the PD-1 receptor present in CD8^+^ T cells (Kalaora et al, 2022). Although not inducing it, PD-1 signaling is critical for maintaining the exhausted state of CD8^+^ T cells (Barber et al, 2006). Blocking PD-1 signaling allows the reinvigoration of effector functions and the expansion of the subpopulation of “exhausted progenitor” CD8^+^ T cells that, unlike their “terminally exhausted” counterparts, maintain polyfunctionality (Miller et al, 2019). Accordingly with these findings, melanoma patients with higher amounts of “depleted progenitor” CD8^+^ T cells respond for a longer time to checkpoint blockade therapy (Miller et al, 2019). Despite these clinical successes, 40–60% of melanoma patients treated with different ICB strategies inhibiting, not only PD-1/PD-L1, but also Cytotoxic T-Lymphocyte Antigen 4 (CTLA-4) or Lymphocyte Activation Gene 3 (LAG-3), even in combination, do not achieve a complete therapeutic response, and several responders undergo relapse (Kalaora et al, 2022). It is therefore essential to thoroughly decipher and understand the mechanisms underpinning ICB resistance, a context in which the different structural, cellular, and molecular components of the tumor microenvironment, including blood vessels and the endothelial cells lining them, play a key role (Kubli et al, 2021).

Autophagy is an evolutionarily conserved process in which, by the formation of double-membrane vesicles, damaged cytosolic molecules, and organelles, as well as pathogens, are selectively isolated and routed toward lysosomal degradation (Vargas et al, 2023). In addition to enabling nutrient recycling and metabolism control, by removing danger-associated molecular patterns (DAMPs) and pathogen-associated molecular patterns (PAMPs) contained in malfunctional molecular aggregates and organelles, and microbes, autophagy also counteracts and curbs inflammation (Deretic, 2021). In the article published in the last issue of *EMBO Molecular Medicine*, Verhoeven and collaborators unveil how the ablation of autophagy promotes the acquisition by tumor endothelial cells (TECs) of a nuclear factor κB (NF-κB)-dependent inflammatory phenotype that, supporting CD8^+^ T-cell infiltration and activity, promotes the response of cutaneous melanoma to ICB therapy with anti-PD-1 (Verhoeven et al, 2023). These findings lend strength and support to the concept that the development and use of autophagy-inhibiting drugs (Whitmarsh-Everiss and Laraia, 2021) in endothelial cells (Verhoeven et al, 2023) and tumor cells (Yamamoto et al, 2020) may be exploited to counteract ICB resistance and increase the efficacy of anti-cancer immunity.

**Figure 1:**
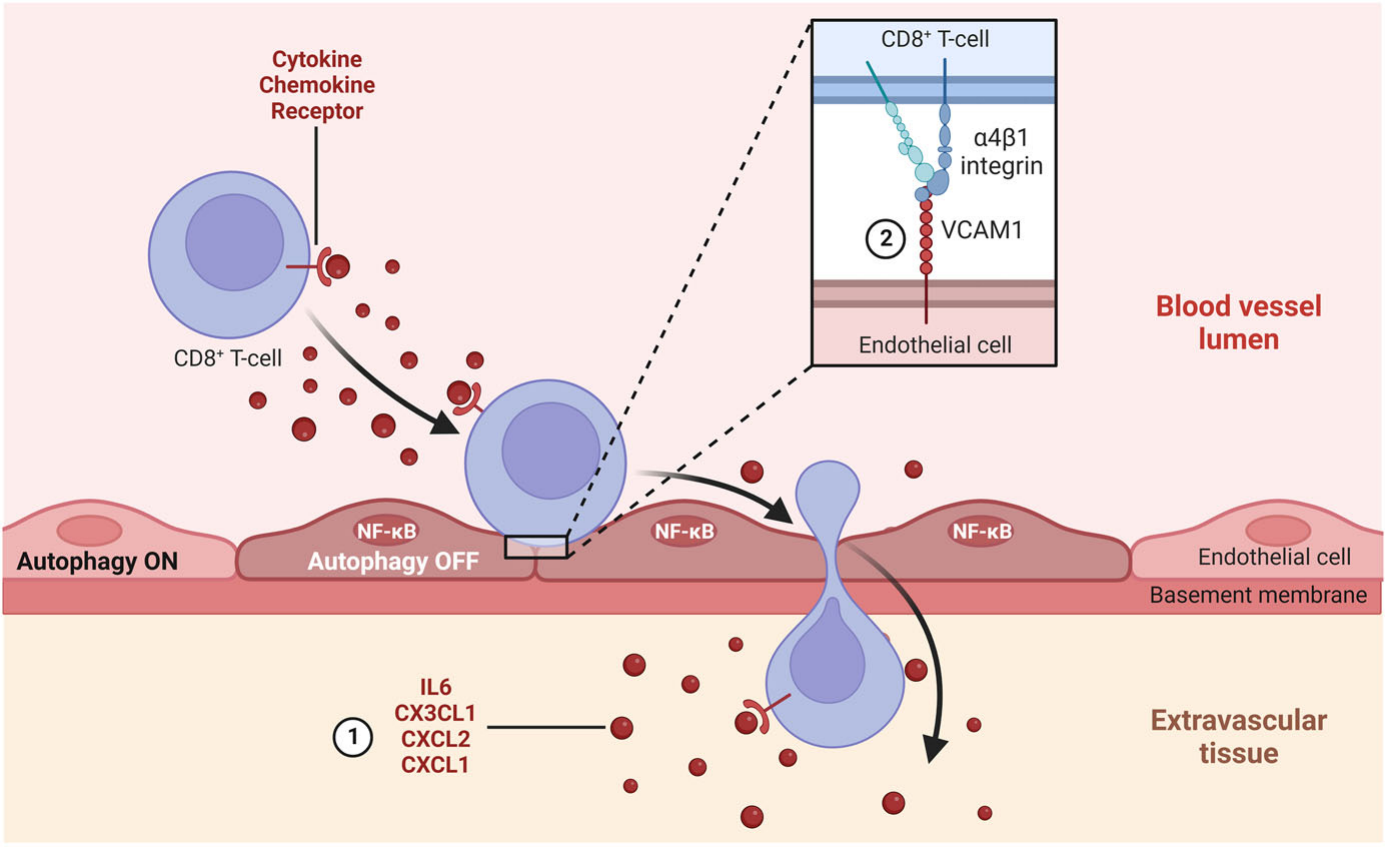
Autophagy inhibition in melanoma blood vessel endothelial cells promotes CD8 + T cell extravasation. Knock-down of key genes controlling the autophagy process promotes, through STING-dependent and independent mechanisms, the translocation of the pro-inflammatory transcription factor NF-κB into the nucleus of tumor endothelial cells. NF-κB promotes the transcription of genes coding for (1) cytokines and chemokines, which, by activating integrin adhesive receptors, attract lymphocytes; (2) transmembrane proteins such as VCAM1 that act as ligands of leukocyte integrins, such as α4β1. As a consequence, CD8^+^ T cells, which more easily exit tumor blood vessel walls in which endothelial autophagy is inhibited, target and eradicate melanoma cells under the stimulation of the anti-PD-1 immune checkpoint blockade therapy. Created with BioRender.

Verhoeven and collaborators first observed how the depletion of autophagy-involved genes (such as *Atg*5, *Atg*12, and *Atg*9) in TECs improves the immunosurveillance status in two syngeneic mouse models of melanoma with different degree of immunogenicity by enhancing the ability to attract Granzyme B (GrZB) protease expressing CD8^+^ T-cells that infiltrate and restrain tumor growth. The authors then revealed how the negative modulation of TEC autophagy fosters a transcriptional, pro-inflammatory program, which increases the expression levels of genes encoding for endothelial cell adhesion molecules (VCAM_1_, ICAM_1_, SELE, and SELP), immune-attracting cytokines or chemokines (IL6, CX_3_CL_1_/fractalkine, CXCL_2_, and CXCL_1_), sets of proteins involved in anti-viral/type 1 IFN response (IFIH_1_, IRF_1_, IRF_7_, TLR_4_), and elements of the antigen presentation machinery (H_2_-K_1_, H_2_T_23_ and H_2_-D_1_). Thus, the inhibition of autophagy in TECs promotes the synthesis of chemoattractants and cell adhesion molecules, which support lymphocyte extravasation (Fig. 1), as well as elements of type 1 IFN responses and antigen presentation, which suggest the acquisition of immunomodulatory functions, including the ability to bolster T-cell function. The murine transcriptome data were confirmed both in terms of expression of mouse TEC surface proteins and human multicancer transcriptome, hence corroborating the concept that, irrespective of the species, TECs with low autophagic function display increased immunomodulatory functions. In keeping with these findings, the authors also discovered that autophagy blockade in TECs of melanoma-bearing mice, other than enhancing the recruitment and activity of tumor-infiltrating lymphocytes, significantly sustains over time the anti-tumor effects of the anti-PD-1 ICB therapy, even without enhancing its efficacy.

Mechanistically, the authors reveal that, although activated by cytosolic double-stranded DNA released from damaged mitochondria, the cytosolic autophagy receptor stimulator of interferon genes (STING) is however dispensable for the acquisition of the NF-κB-dependent inflammatory phenotype observed in autophagy-depleted endothelial cells, both in vitro and in vivo. The latter finding points out to the existence of an alternative, yet to be discovered, STING-independent mechanism of canonical (RELA-dependent) and non-canonical (RELB-dependent) NF-κB activation following endothelial autophagy loss. In this regard, autophagy has, for example, been described to foster RELA degradation and thus counteract canonical NF-κB signaling in tumor-associated macrophages in vivo (Monkkonen and Debnath, 2018). Finally, further validating their in vivo and in vitro findings, Verhoeven and colleagues identified an enriched, before treatment, Inflammatory^high^/Autophagy^low^ TEC signature in melanoma patients that subsequently responded to anti-PD-1 immunotherapy. Accordingly, multiplex immunohistochemistry analysis of the same responding melanoma patients unveiled VCAM1^+^ TEC-lined inflamed blood vessels with increased infiltrating naive (PD-1^−^/GrZB) and activated/effector (PD-1^+^/GrZB^+^) CD8^+^ T-cells laying in their proximity (Fig. 1).

In sum, the work of Verhoeven and collaborators unveils for the first time that in TECs, autophagy exerts an anti-inflammatory function that significantly limits the efficacy of the anti-tumor immune response in melanoma treated with anti-PD-1 ICB therapy. These findings pave the way for the implementation of potential new combo therapies in which autophagy inhibiting and ICB drugs may be simultaneously administered, at least in melanoma. Moreover, it must be considered that in other tumor histotypes autophagy inhibition or activation of the NF-κB-dependent inflammatory response have been reported to promote tumor progression (Monkkonen and Debnath, 2018). Therefore, further work is needed to better grasp the tissue, cellular, and molecular context in which pharmacological inhibition of autophagy and activation of NF-κB-regulated inflammation may be efficiently and optimally harnessed to enhance the benefit of ICB therapy. In this view, for example, the identification of signaling pathways specifically controlling autophagy function and/or activation of canonical and noncanonical NF-κB signaling in TECs may allow to verify whether the novel mechanisms that Verhoeven and collaborators have shown to be therapeutically exploitable to support immunotherapy in melanoma may also be applied in other cancer settings, including those in which the global inhibition of autophagy has been reported to sustain tumor progression.
